# Memory decline in young stroke survivors during a 9-year follow-up: A cohort study

**DOI:** 10.3389/fneur.2022.1069686

**Published:** 2022-11-25

**Authors:** Siiri Laari, Katri Turunen, Tatu Kauranen, Satu Mustanoja, Marius Lahti-Pulkkinen, Turgut Tatlisumak, Erja Poutiainen

**Affiliations:** ^1^Department of Psychology and Logopedics, Faculty of Medicine, University of Helsinki, Helsinki, Finland; ^2^HUS Neurocenter, Helsinki University Hospital and University of Helsinki, Helsinki, Finland; ^3^Department of Neurology and Clinical Neurophysiology, Lapland Central Hospital, Rovaniemi, Finland; ^4^Institute of Neuroscience and Physiology, Sahlgrenska Academy at University of Gothenburg, Gothenburg, Sweden; ^5^Department of Neurology, Sahlgrenska University Hospital, Gothenburg, Sweden

**Keywords:** memory decline, young stroke survivor, ischemic stroke, long-term cognitive outcome, cognitive impairment, follow-up

## Abstract

**Introduction:**

A decade after stroke, young stroke survivors continue to suffer from cognitive impairment. However, it is not known whether this long-term cognitive outcome is caused in part by further cognitive decline or solely by incomplete recovery from the acute effects of ischemic stroke. We studied changes in three cognitive domains over a 9-year follow-up period after first-ever and only ischemic stroke.

**Patients and methods:**

In this prospective, two-center cohort study, we recruited consecutive 18–65 year-old patients with acute stroke between 2007 and 2009, along with demographically matched stroke-free controls. We performed comprehensive neuropsychological assessments at 3 months, 2, and 9 years after stroke, and we also performed neurological examinations at the time of inclusion and at the 9-year follow-up. We assessed the associations among stroke, follow-up time and long-term cognitive outcomes using repeated-measures analysis of variance.

**Results:**

The subjects comprised 85 patients who had had their first-ever and only ischemic stroke (mean age 53 years at inclusion), along with 31 stroke-free demographic controls. We compared the cognitive changes in patients to those in controls over a 9-year follow-up. After initial recovery between 3 months and 2 years after stroke, patients showed a decline in memory between 2 and 9 years after stroke compared to controls within the same time interval (immediate recall *p* < 0.001; delayed recall *p* < 0.001; list learning *p* < 0.001). Other than memory, we found no difference in cognitive changes between poststroke patients and controls.

**Discussion:**

Our main finding was memory decline over a decade in young first-ever stroke patients with no further stroke or neurodegenerative disease. Our study extends the previous results of further memory decline in elderly stroke survivors to young stroke survivors.

**Conclusion:**

Young stroke survivors might be at risk of memory decline over the decade following the stroke.

## Introduction

The incidence of ischemic stroke has increased among working-age adults with many productive years ahead (1, 2). Encouragingly, recent advances in acute ischemic stroke treatment now allow more patients to achieve functional independence (3). By an increasing margin, cognitive impairment is the leading symptom that hinders young stroke survivors from returning to work (4, 5). Nonetheless, remarkably few studies have assessed the long-term cognitive prognosis of young stroke survivors.

Young stroke survivors suffer from long-term cognitive impairment at 10 years after stroke, as previous cross-sectional studies have shown (6–8). However, it is impossible to determine from these cross-sectional studies whether incomplete recovery from the acute effects of stroke fully explains this long-term cognitive impairment. Alternatively, cognition might further decline over the course of the following years, as seen in longitudinal studies of elderly stroke survivors (9–12). However, older age increases cognitive risks during a ten-year-follow-up after stroke (10). Young brains may be able to compensate for brain damage more effectively than elderly brains (13). A small, pioneering study in young stroke survivors found improvement in working memory and a decline in visuomotor speed over the 10 years following the stroke (14). However, no previous longitudinal study has been large enough to achieve high statistical power and included a stroke-free comparison group to control for the effect of normal aging on cognition.

In this prospective cohort study, we compared changes in three cognitive domains between young first-ever ischemic stroke patients and their stroke-free demographic controls over a 9-year follow-up period.

## Materials and methods

### Young stroke cohort and follow-up study design

Patients were part of a consecutive stroke inpatient cohort recruited from Helsinki University Central Hospital and Lapland Central Hospital from April 2007 to October 2009. The inclusion criteria were a first-ever diagnosis of supratentorial ischemic stroke, age 18–65 years, no severely altered state of consciousness or relevant neurological or psychiatric history or comorbidity, and Finnish as a native language. Demographically comparable controls—all of whom were patients' spouses or relatives—met all the criteria set for patients except for stroke.

In this study, we included only those participants who completed all three follow-up visits. Patients were prospectively followed up at 3 months, 2, and 9 years after the index event (controls were followed up first at a 3-month interval and then at a 9-year interval from the first visit). The Ethics Committee of Helsinki University Central Hospital approved the study and consent procedure (approval number 356/2017), and all participants signed an informed consent form.

### Ischemic stroke data

Neurological data to confirm the predictive variable of stroke diagnosis as well as potential confounders and effect modifiers were collected at the acute care hospital. An experienced stroke neurologist specified ischemic supratentorial stroke diagnoses with brain imaging and neurological measures. All patients underwent brain imaging within the first days after stroke with magnetic resonance imaging (MRI) and/or brain computed tomography (CT). The neurologist evaluated the lesion size (in millimeters from the plane in which the largest diameter was observed), lesion locations and age-related white matter changes (ARWMCs) according to a widely used scale (15). The neurologist used the Trial of Org 10172 in Acute Stroke Treatment (TOAST) criteria (16) to determine the stroke etiology (large-artery atherosclerosis, cardioembolic, small-artery occlusion, other determined, or undetermined), the Barthel Index (17) to evaluate physical functional status, and the National Institutes of Health Stroke Scale (NIHSS) (18) to measure the severity of stroke at the time of discharge from the acute care hospital. Stroke risk factors were collected from the patient's case history and included in the structured interviews during acute care. The Barthel Index, NIHSS, and structured interviews for stroke risk factors were repeated at the 9-year follow-up. Controls gave structured interviews similar to those conducted with patients at their first and last follow-up visits.

### Long-term cognitive outcomes

To compare cognitive outcomes between patients and controls, an experienced neuropsychologist conducted an assessment according to a strict protocol at each of the three follow-up visits. Assessment included 10 validated cognitive tests covering three cognitive domains: *Memory* comprised the Logical Memory Test I and II (immediate and delayed recall of a story) subtests of the Wechsler Memory Scale-Revised (WMS-R) (19) and the 10-word list-learning task (20). *Executive function* comprised the phonemic fluency task; the Trail Making Test, difference score of parts B and A (21); the Stroop test, difference score of the interference and naming parts (22); and the Digit Span and Digit Symbol subtests of the Wechsler Adult Intelligence Scale-Third Edition (WAIS-III) (23). *Reasoning* comprised the Similarities and Block Design subtests of the WAIS-III (23). The participants' mood state was assessed with the modified Profile of Mood States (POMS) questionnaire at every follow-up visit (24).

### Statistical methods

SPSS version 22 (IBM, Armonk, NY, USA) software was used for statistical analyses. Basic transformations (rank order, logarithmic, square root) were used when necessary to account for skewness and to obtain normal distributions. Missing values were not replaced. The demographic and clinical variables were compared using an independent *t-*test and a chi-square (χ^2^) test between patients and controls and between patients who were included and those who dropped out.

In the main analyses, our statistical design entailed a follow-up study at three time points: 3 months, 2, and 9 years poststroke. We compared the change in stroke patients' cognitive test performance to the change in controls' performance between follow-up assessments. We performed two-way repeated measures analysis of variance (ANOVA) for each cognitive test with follow-up time as the repeated within-subject factor and stroke (stroke vs. no stroke) as the predictor variable. Second, we incorporated those potential confounders that differed between patients and controls and had enough cases per covariate group to enable reliable analyses into the model. We used Greenhouse-Geisser correction in cases of sphericity violations. When ANOVA indicated time^*^stroke interactions, we compared differences between groups with an independent samples *t-*test and within groups with a dependent samples *t-*test. After Bonferroni adjustment, the significance level was set at 0.005 (0.05/10) to correct for multiple comparisons in the main analyses.

Finally, in secondary analyses, we compared the change in memory test performance of patients with cortical lesions to the change in performance of patients with subcortical lesions between follow-up assessments. We performed two-way repeated measures analysis of variance (ANOVA) for each three memory tests with follow-up time as the repeated within-subject factor and location (cortical vs. subcortical) as the predictor variable. Similarly, we compared the change in memory of patients with left vs. right lesions.

## Results

### Ischemic stroke, demographics and clinical characteristics

**Figure 1:**
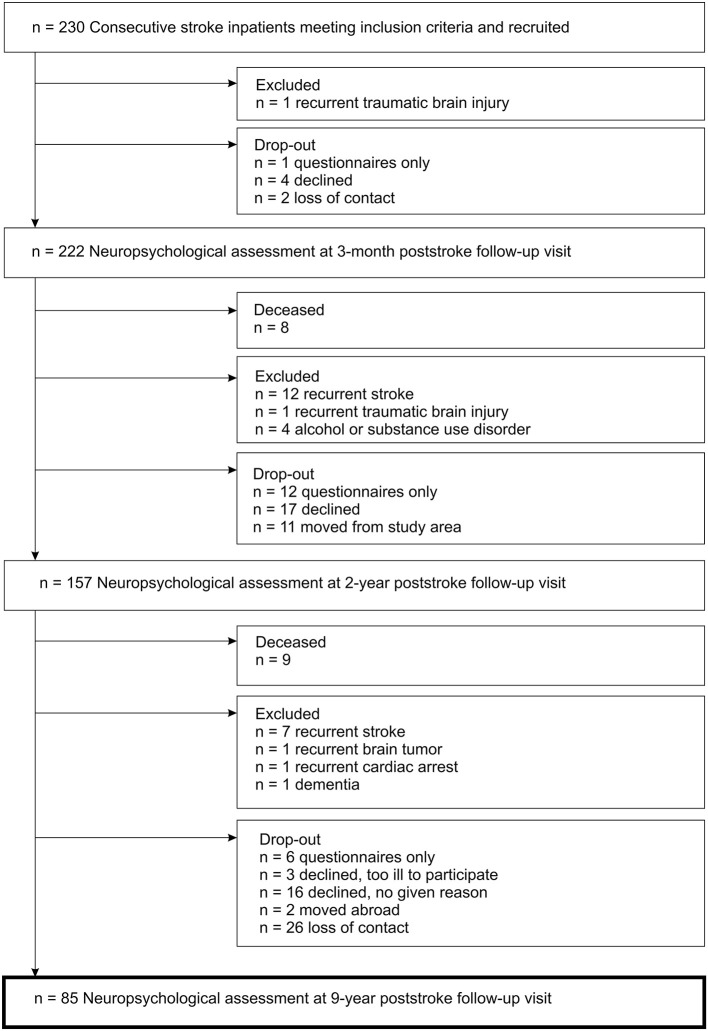
Flow diagram of the inclusion process for the study cohort with first-ever ischemic stroke.

Ultimately, 85 stroke patients (Figure 1) and 31 stroke-free controls were included in the follow-up study. Ischemic stroke characteristics of patients included in this study are presented in Table 1.

**Table 1:** Ischemic stroke characteristics at the time of the incident stroke for 85 stroke patients included in this study.

NIH Stroke Scale at discharge
0 points, no deficit	26 (30.6)
1–6 points	56 (65.9)
7 or more points	3 (3.5)
Barthel Index impaired, 0–95 points	7 (8.8)
Infarct size, largest diameter in mm	22.9 (24.5)
Silent infarctions	20 (23.8)
Infarct side
Nonvisible	17 (20.2)
Left	35 (41.7)
Right	27 (32.1)
Bilateral	5 (6.0)
Infarct location
Nonvisible	17 (20.2)
Cortical	26 (30.6)
Subcortical	25 (29.4)
Cortico-Subcortical	16 (18.8)
Infarct location
Frontal	3 (3.5)
Parietal	8 (9.4)
Temporal	7 (8.2)
Occipital	8 (9.4)
Basal ganglia	24 (28.2)
Several locations	21 (24.7)
Stroke etiology by TOAST criteria
Large-artery atherosclerosis	18 (21.2)
Cardioembolism	18 (21.2)
Small-artery occlusion	16 (18.8)
Other determined etiologies	12 (14.1)
Undetermined etiologies	21 (24.7)
White matter changes	21 (25.0)

NIH Stroke Scale, National Institutes of Health Stroke Scale at discharge; TOAST, the Trial of Org 10172 in Acute Stroke Treatment.

Goldstein et al. (18).

Mahoney and Barthel (17).

Adams et al. (25).

There were no differences in demographic variables between stroke patients and controls (Table 2). The average age of the patients was 53 years at the time of inclusion, and the average age of the controls was 54 years. The mean follow-up duration was 9 years.

**Table 2:** Comparison of demographic and clinical characteristics between ischemic stroke patients and stroke-free controls.

	**Patients** ***n =* 85**	**Controls** ***n =* 31**	***P*-value**
**At the time of the inclusion**			
Age^†^	53.2 (10.7)	54.1 (8.7)	0.751
Sex, men^‡^	53 (62.4)	21 (67.7)	0.593
Education, years^†^	12.6 (2.7)	13.2 (3.0)	0.343
Barthel Index, impaired^‡^ (17)	7 (8.8)	0 (0.0)	0.089
Diabetes mellitus^‡^	10 (11.8)	1 (3.2)	0.165
High serum cholesterol^‡^	47 (56.0)	17 (54.8)	0.915
High blood pressure^‡^	54 (63.5)	18 (58.1)	0.591
Atrial fibrillation^‡^	11 (12.9)	0 (0.0)	0.030*
Coronary artery disease^‡^	7 (8.2)	0 (0.0)	0.099
Myocardial infarction^‡^	4 (4.7)	0 (0.0)	0.219
Cardiac failure^‡^	3 (3.5)	0 (0.0)	0.209
Cigarette smoking^‡^	26 (30.6)	4 (12.9)	0.054
Alcohol doses weekly^†^	5.8 (7.8)	3.4 (4.5)	0.114
Mood state, POMS sum^†^ (2)	42.9 (22.4)	26.6 (14.4)	< 0.001***
**At the 9-year follow-up**			
Barthel Index impaired^‡^ (1)	14 (16.5)	3 (9.7)	0.360
ADL difficulties^‡^	15 (18.3)	5 (16.1)	0.788
Diabetes mellitus^‡^	19 (22.4)	2 (6.5)	0.049*
High serum cholesterol^‡^	53 (62.4)	10 (32.3)	0.004*
High blood pressure^‡^	52 (61.2)	12 (38.7)	0.031*
Atrial fibrillation^‡^	18 (21.2)	1 (3.2)	0.021*
Coronary artery disease^‡^	13 (15.3)	0 (0.0)	0.021*
Myocardial infarction^‡^	6 (7.1)	0 (0.0)	0.129
Cardiac failure^‡^	6 (7.1)	1 (3.2)	0.443
Cigarette smoking^‡^	16 (18.8)	2 (6.5)	0.103
Alcohol doses weekly^†^	3.6 (5.8)	3.6 (4.5)	0.997
BMI over 25^‡^	43 (50.6)	10 (32.3)	0.120
Mood state, POMS sum^†^ (23)	34.8 (19.7)	29.6 (14.3)	0.240

*** Significant at *p* < 0.001, *significant at *p* < 0.05.

^†^Compared with the t-test, mean (SD),^‡^Compared with the chi-square (χ^2^) test, n (%).

Barthel index impaired, 0–95 points; *Stroke risk factor data are self-reported in structured interviews:* High serum cholesterol is either total cholesterol ≥5 mmol/l or LDL ≥3 mmol/l; High blood pressure ≥140/85 mmHg; Mood state was assessed with the sum score (the higher score is, the more negative mood) of the modified Profile of Mood States (POMS) questionnaire; BMI, Body mass index.

Regarding clinical characteristics, stroke patients had more atrial fibrillation and lower mood state at the time of the incident stroke compared to controls (Table 2). Further, patients had more often diabetes and coronary artery disease, more atrial fibrillation and higher serum cholesterol, and higher blood pressure levels at 9 years poststroke than controls (Table 2).

There were no differences in stroke characteristics (such as stroke severity, lesion size or lesion location) between the 85 patients included in this study and the 100 patients who dropped out of 9-year follow-up; however, the included patients were significantly more educated than those who dropped out (12.6 vs. 11.6 years of education; *p* = 0.011).

To measure the long-term cognitive outcome of stroke, we compared the change in patient performance on each cognitive test to the change in control performance between three follow-up assessments.

### Memory decline

Regarding the main effect of stroke, patients performed worse on the list-learning task than controls across all follow-up points. Regarding the main effect of follow-up time, performance on immediate and delayed recall improved between 3 months and 2 years in all participants and subsequently declined between 2 and 9 years (Supplementary Table I). Most importantly, the interaction between stroke and follow-up time qualified these main effects as follows.

**Figure 2:**
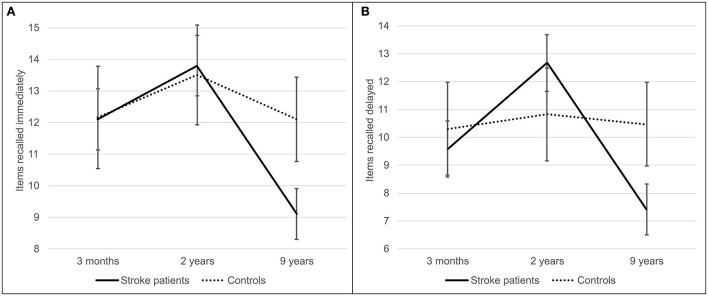
Immediate **(A)** and delayed **(B)** recall of a story from the logical memory test component of the wechsler memory scale. Stroke patients (solid line) recalled items at three follow-up time points–3 months, 2, and 9 years poststroke—and were compared to stroke-free controls (dashed line). Error bars represent 95% confidence intervals.

Memory decline between 2 and 9 years was greater in stroke patients than in controls (Figure 2), as indicated by the interaction effect between stroke and follow-up time for immediate [*F*_(2, 228)_ = 12.31, *p* < 0.001, $\eta_{p}^{2}$ = 0.097] and delayed recall [*F*_(2, 226)_ = 19.61, *p* < 0.001, $\eta_{p}^{2}$ = 0.148] and list learning [*F*_(2, 228)_ = 7.52, *p* = 0.001, $\eta_{p}^{2}$ = 0.062]. The subsequent interaction effects confirmed that patients, but not controls, performed worse at 9 years than at 3 months in immediate recall [*F*_(1, 114)_ = 15.54, *p* < 0.001, $\eta_{p}^{2}$ = 0.120] and delayed recall [*F*_(1, 113)_ = 9.20, *p* = 0.003, $\eta_{p}^{2}$ = 0.075] and worse at 9 years than at 2 years in immediate recall [*F*_(1, 114)_ = 23.15, *p* < 0.001, $\eta_{p}^{2}$ = 0.169], delayed recall [*F*_(1, 113)_ = 41.12, *p* < 0.001, $\eta_{p}^{2}$ = 0.267] and list learning [*F*_(1, 114)_ = 8.46, *p* = 0.004, $\eta_{p}^{2}$ = 0.069]. In addition, patients but not controls performed better at 2 years than at 3 months in delayed recall [*F*_(1, 113)_ = 9.95, *p* = 0.002, $\eta_{p}^{2}$ = 0.081].

*Post hoc* comparisons confirmed the interpretation of the stroke^*^follow-up time interaction: controls outperformed patients at 9 years poststroke in all three memory tests (*t*_(114)_ = −3.78, *p* < 0.001; *t*_(114)_ = −3.42, *p* = 0.001; *t*_(114)_ = −3.23, *p* = 0.002], and patients recalled significantly fewer items of a story at 9 years poststroke than at 3 months [*t*_(84)_ = 7.68, *p* < 0.001; *t*_(83)_ = 6.19, *p* < 0.001].

Finally, to control the stroke^*^follow-up time interaction results for confounding factors that differed between patients and controls, we added mood state, hypertension, and hypercholesterolemia to the analysis. Adding these covariates did not change the interaction effects between stroke and follow-up time. Moreover, excluding patients who had transient ischemic attacks during follow-up did not change the interaction effects.

As stated, we also did additional secondary analyses to study the effect of lesion location on memory change. First, we compared the memory change between patients with subcortical lesions (*n* = 25) and patients with cortical lesions (*n* = 26) between three follow-up assessments. The interaction effects between only subcortical or only cortical lesion location and follow-up time were not significant either for immediate recall [*F*_(2, 98)_ = 7.96, *p* = 0.264, $\eta_{p}^{2}$ = 0.027], delayed recall [*F*_(2, 96)_ = 0.89, *p* = 0.413, $\eta_{p}^{2}$ = 0.018] or list learning [*F*_(2, 98)_ = 2.55, *p* = 0.083, $\eta_{p}^{2}$ = 0.049]. Second, we compared the memory change between patients with left sided lesions (*n* = 35) and patients with right sided lesions (*n* = 27) between three follow-up assessments. The interaction effects between only left sided or only right sided lesion location and follow-up time were not significant either for immediate recall [*F*_(2, 120)_ = 5.47, *p* = 0.005, $\eta_{p}^{2}$ = 0.084], delayed recall [*F*_(2, 118)_ = 4.14, *p* = 0.018, $\eta_{p}^{2}$ = 0.066] or list learning [*F*_(2, 120)_ = 0.49, *p* = 0.612, $\eta_{p}^{2}$ = 0.008].

### Executive function

Regarding the main effect of stroke, patients performed worse than controls on the Trail Making Test and on the Digit Span and Digit Symbol subtests of the WAIS-III across all follow-up points (Supplementary Table I). Regarding the main effect of follow-up time, the performance of both patients and controls worsened over time on the Trail Making Test, the phonemic fluency task, and the Digit Span and Digit Symbol subtests of the WAIS-III.

### Reasoning

Regarding the main effect of follow-up time, the performance of both patients and controls worsened over time in the Block Design subtest of the WAIS-III (Supplementary Table I).

## Discussion

We compared changes in three cognitive domains between a consecutive cohort of young first-ever stroke patients and a cohort of stroke-free controls over a 9-year follow-up period. The main finding was memory decline over the course of a decade in young first-ever stroke patients who did not experience a further stroke or neurodegenerative disease. Except for memory, we found no difference in cognitive change between poststroke patients and controls. Additionally, stroke and aging as separate variables had negative associations with performance in all three cognitive domains, as could be excepted.

The main finding was memory decline associated with first-ever and only stroke between the 2-year and 9-year follow-ups after initial recovery by 2 years poststroke. Decline was significant in all three tests of memory performance: immediate recall of a story, delayed recall of a story and list learning. Stroke patients' performance in immediate and delayed recall of a story was more impaired at 9 years than at either 3 months or 2 years poststroke. Moreover, this memory decline during follow-up was greater in stroke patients than in demographic controls; thus, aging alone does not explain the decline. Furthermore, the memory performance of patients was more impaired at 9 years poststroke than at 3 months poststroke. In light of the present results, memory impairment a decade after stroke as seen in previous cross-sectional studies (6, 26) might be due to a further decline and not only to incomplete recovery from the initial stroke. Altogether, our findings are consistent with previous longitudinal studies that reported memory decline over 3–12 years of follow-up in older stroke survivors (11, 12). Our study builds on these previous findings of memory decline in elderly stroke survivors by extending them to young stroke survivors.

Ischemic stroke might cause memory decline for various reasons. One possible reason is the risk of neurodegenerative disease, which increases with age. Regarding the highest end of the age rage 18–65 years in our study, underlying neurodegenerative process might underlie the memory decline observed in our study. Average age of the patients in our study was 53 years at inclusion, which is, although comparable to a pioneering longitudinal study in young stroke survivors (14), yet bit higher compared to several studies on young stroke (6–8). Considering the memory decline we observed, the most likely underlying neurodegenerative disease might be Alzheimer's disease, in which memory impairment is most often the leading symptom. Stroke is suggested to be involved in the development of Alzheimer's disease through overlapping pathologies or by enhancing the degenerative effect of neuronal tissue loss caused by amyloid and tau pathologies (27). Thus, although none of the patients in our study had neurodegenerative disease diagnosed nor reported more difficulties in activities of daily living than those in the control group, it is possible that subclinical Alzheimer's disease could partly explain the memory decline observed in our study.

Alternatively, vascular risk factors may cause ongoing cerebrovascular injury accompanied by cognitive decline (28, 29). However, cerebrovascular injury typically has a stronger association with executive function and processing speed than with memory (28, 29). As would be expected, stroke had an overall negative effect on executive function in our study. However, we found no difference in executive function change between poststroke patients and controls, contrary to the post stroke memory decline over the course of a decade. Instead, executive function declined in both patients and controls during the 9-year follow-up, which is consistent with the effects of normal aging (30). Furthermore, excluding participants who had recurrent transient ischemic attacks during follow-up or adjusting analyses for hypertension and hypercholesterolemia did not change the results of the present study. Altogether, it is unlikely that vascular risk factors would explain the memory decline observed.

Another explanation for memory decline could be stroke-induced neuronal loss remote from the acute lesion (31, 32). According to recent studies in young stroke survivors, long-term neuronal loss can degrade white matter integrity or cause hippocampal atrophy remote from the lesion (31, 33). Ischemic stroke lesions outside the hippocampus are associated with smaller ipsilateral hippocampal volumes 10 years poststroke, which in turn are associated with impaired memory performance (33). Our small number of patients with pure temporal lesions precluded the possibility to reliably study more specific brain-behavior relations regarding observed memory decline. However, we found no differences in memory decline over time related to lesion location, neither between patients with only cortical and only subcortical lesions nor between those with left and right sided lesions. In the future, repeating this study with larger sample size and greater focus on brain imaging with MRI at several follow-up points could produce interesting findings that account more for brain-behavior relations. Furthermore, combining memory task measured with functional MRI to the follow-up study could identify the mechanisms linking incident ischemic stroke to memory decline over the following decade.

### Strengths and limitations

Our study has several strengths. We assessed the same groups of stroke patients and healthy controls at three time points, whose intervals were similar between groups. Comprehensive neuropsychological assessment at all three time points enabled us to determine the temporal trajectories of cognitive domains. With a corresponding follow-up of the healthy control group, we were able to adjust the observed cognitive changes for the normal effects of aging. We had a sufficient sample size to answer the research questions.

An unfortunate limitation of this study was the high dropout rate, although the rate was not exceptional for a long-term follow-up study. Moreover, there were no differences in stroke characteristics between the included and drop-out patients. Our sample is representative of the original consecutive cohort except that the patients who dropped out of the study had less education than the included patients. Low education has been shown to increase cognitive risks during the ten-year poststroke period (10). Thus, it is possible that long-term cognitive decline after stroke was underestimated in our study. In addition, generalizability of our findings is limited in terms of age. With respect to the emphasis on older end of the young stroke population, our results may not be applicable to patients at the lower end of the age spectrum. Furthermore, we adjusted our analysis only for those stroke risk factors that had enough cases per covariate group. Finally, we could not control for recurrent silent infarctions because we performed brain imaging only at the time of the first-ever stroke. However, the diagnosis of any recurrent brain damage was an exclusion criterion.

## Conclusion

Our findings suggest that ischemic stroke poses a risk of further memory decline in young stroke survivors during the decade after the stroke, as previously shown in elderly stroke survivors. The high risk of memory decline in the long term warrants further study to optimize treatments and rehabilitation for these young patients.

## Data availability statement

The datasets presented in this article are not readily available because of legislative issues regarding patient privacy. Requests to access the datasets should be directed to SL, siiri.laari@hus.fi.

## Ethics statement

The studies involving human participants were reviewed and approved by Ethics Committee of Medical Research, Helsinki University Hospital and University of Helsinki, Biomedicum 2 Tukholmankatu 8 B (PL 20) 00014 Helsinki University, Finland. The patients/participants provided their written informed consent to participate in this study.

## Author contributions

EP and TT researched the literature, conceived the study, and obtained the ethical approval. SL, KT, TK, SM, and EP were involved in protocol development, patient recruitment, and interpretation of data. SL and ML-P were involved in data analysis. SL wrote the first draft of the manuscript. All authors reviewed and edited the manuscript and approved the final version.

## Funding

This study was supported by a grant (37/26/2007) from the Finnish Social Insurance Institution's Research Department, Helsinki University Hospital and University of Helsinki, Finland, and by a grant (330206) from the Academy of Finland. The funding sources were not involved in the study design, interpretation of data, or writing of the article.

## Conflict of interest

The authors declare that the research was conducted in the absence of any commercial or financial relationships that could be construed as a potential conflict of interest.

## Publisher's note

All claims expressed in this article are solely those of the authors and do not necessarily represent those of their affiliated organizations, or those of the publisher, the editors and the reviewers. Any product that may be evaluated in this article, or claim that may be made by its manufacturer, is not guaranteed or endorsed by the publisher.
