# Supplementary material for: Memory decline in young stroke survivors during a 9-year follow-up: A cohort study
Source: Front Neurol. 2022 Nov 25;13:1069686. doi: 10.3389/fneur.2022.1069686 (PMC9732240; doi:10.3389/fneur.2022.1069686)
Supplement: Supplementary file 1 [file Table_1.DOCX]

Supplementary Table I. Main effects of stroke and follow-up time on cognitive performance of ischemic stroke patients and stroke-free controls at three

follow-up assessments compared with the repeated-measures ANOVA. Unadjusted estimates with confidence intervals (CI) at three follow-ups.

|  | Stroke patients, N = 85, mean (CI) | | | Stroke-free controls, N = 31, mean (CI) | | | Stroke  F_1,114_ | | Follow-up time F_2,228_ | |
| --- | --- | --- | --- | --- | --- | --- | --- | --- | --- | --- |
|  | 1^st^ follow-up | 2^nd^ follow-up | 3^rd^ follow-up | 1^st^ follow-up | 2^nd^ follow-up | 3^rd^ follow-up | P | η_p_^2^ | P | η_p_^2^ |
| **Memory** |  |  |  |  |  |  |  |  |  |  |
| WMS LM I Immediate | 12 (11-13) | 14 (13-15) | 9 (8-10) | 12 (11-14) | 14 (12-15) | 12 (11-13) | 0.249 | 0.012 | 0.001 | 0.237 |
| WMS LM II Delayed† | 9 (8-10) | 12 (11-13) | 7 (6-8) | 10 (8-12) | 10 (9-12) | 10 (9-12) | 0.444 | 0.005 | <0.001 | 0.189 |
| 10-word list learning‡ | 36 (34-38) | 41 (39-42) | 37 (35-39) | 42 (39-46) | 43 (40-46) | 42 (39-45) | 0.002 | 0.078 | 0.025 | 0.032 |
| **Executive function** |  |  |  |  |  |  |  |  |  |  |
| Phonemic fluency† | 13 (12-14) | 17 (15-18) | 15 (14-16) | 15 (13-17) | 18 (16-20) | 18 (16-20) | 0.046 | 0.035 | <0.001 | 0.187 |
| Trail Making Test (R)§ | 72 (62-82) | 66 (57-75) | 81 (71-91) | 45 (29-62) | 39 (23-55) | 56 (39-72) | 0.002 | 0.085 | <0.001 | 0.127 |
| Stroop Test (R)¶ | 53 (47-59) | 48 (43-53) | 57 (51-63) | 44 (34-55) | 39 (31-48) | 42 (32-53) | 0.138 | 0.019 | 0.025 | 0.032 |
| WAIS-III Digit Span | 6.2 (5.7-6.7) | 6.7 (6.2-7.2) | 6.2 (5.7-6.7) | 7.6 (6.7-8.4) | 8.3 (7.4-9.1) | 7.4 (0.4) | 0.003 | 0.076 | <0.001 | 0.073 |
| WAIS-III Digit Symbol | 54 (50-57) | 59 (55-63) | 50 (46-54) | 68 (61-74) | 70 (64-77) | 62 (55-69) | 0.001 | 0.089 | <0.001 | 0.317 |
| **Reasoning** |  |  |  |  |  |  |  |  |  |  |
| WAIS-III Block Design | 39 (36-42) | 40 (38-43) | 35 (33-38) | 44 (39-48) | 45 (41-50) | 40 (36-45) | 0.064 | 0.030 | <0.001 | 0.131 |
| WAIS-III Similarities† | 22 (20-23) | 23 (21-24) | 21 (20-22) | 24 (21-26) | 25 (23-26) | 24 (22-26) | 0.017 | 0.049 | 0.085 | 0.021 |

† Missing values n = 1 ‡ A rank-order transformation was used in the analysis. § A logarithmic transformation was used in the analysis. ¶A square-root transformation was used in the analysis. WMS = Wechsler Memory Scale; LM I Immediate = Logical Memory I, Immediate Recall; LM II Delayed = Logical Memory II, Delayed Recall); Trail Making Test = difference score of parts B and A; Stroop Test = difference score of interference and naming parts; WAIS-III = Wechsler Adult Intelligence Scale. Reverse-scored items, presented as time in seconds, are denoted with an (R). For stroke patients, the 1^st^ follow-up took place at 3 months, the 2^nd^ follow-up at 2 years and the 3^rd^ follow-up at 9 years poststroke. For healthy controls, the 2^nd^ follow-up 3 months after the 1^st^ one, and the 3^rd^ took place 9 years after the 1^st^.
